# The Protective Effect of Naringenin-Oxime on Cisplatin-Induced Toxicity in Rats

**DOI:** 10.1155/2017/9478958

**Published:** 2017-08-28

**Authors:** Ismail Koyuncu, Abdurrahim Kocyigit, Ataman Gonel, Erkan Arslan, Mustafa Durgun

**Affiliations:** ^1^Department of Biochemistry, Faculty of Medicine, Harran University, Sanliurfa, Turkey; ^2^Department of Medical Biochemistry, Faculty of Medicine, Bezmialem Vakif University, Istanbul, Turkey; ^3^Department of Urology, Faculty of Medicine, Harran University, Sanliurfa, Turkey; ^4^Department of Chemistry, Faculty of Science and Literature, Harran University, Sanliurfa, Turkey

## Abstract

The aim of this study is to examine the protective effect of naringenin-oxime (NOX) on cisplatin-induced major organ toxicity and DNA damage in rats. Thirty-five male Wistar albino rats were equally split into five groups as follows: control (i.p., 0.1 ml of saline), Cis administration (i.p., 7 mg/kg b.w.), NOX treatment (i.p., 20 mg/kg b.w., daily for ten days), Cis + NOX20, and Cis + NOX40 combination (i.p., 20 and 40 mg/kg b.w., daily for ten days). Serum and peripheral blood mononuclear leukocytes (PBMC) were obtained from blood. Malondialdehyde, glutathione, total antioxidant and oxidant status, and catalase were measured in serum, liver, and kidney, and oxidative stress index was calculated. In parallel, paraoxonase and arylesterase activities were tested in liver and serum. We used 8-OHdOG as a marker for DNA damage in serum via ELISA and in PMBC via comet assay. Treatment with Cis elevated the levels of serum biochemical parameters, oxidative stress, and DNA damage. Pretreatments of NOX restored biochemical and oxidative stress parameters in serum, renal, and liver tissues (*p* < 0.01) and reduced 8-OHdG level, a finding further supported by comet assay in PBMC. Observations of the present study support the fact that treatment with NOX prevents Cis-induced hepatotoxicity, nephrotoxicity, and genotoxicity by restoring antioxidant system.

## 1. Introduction

Cisplatin (Cis) is a widely used chemotherapeutic agent in treatment regimens for some human malignant tumors [1, 2]. Clinical use of Cis was restricted because of some serious adverse effects such as nephrotoxicity, neurotoxicity, and ototoxicity which greatly hamper its chemotherapeutic efficacy [3–7]. Cis induces cytotoxicity in healthy tissues by contributing to the production of reactive oxygen species (ROS), inducing mitochondrial oxidative harm, inhibiting antioxidant enzymes, and releasing free radicals [8]. The products of free oxygen radicals in cells are responsible to be a significant pathogenic phase and that is supported by enough evidence [9, 10]. Increase in free radical production and decrease in antioxidant defense systems, involving antioxidant enzymes and nonenzymatic molecule called glutathione (GSH), have been shown to mediate the toxicity caused by Cis [11–13]. Those decrements may cause impairment in antioxidant defense mechanisms securing tissue harm, which are induced by free radicals, and mutagenic capacity of Cis. Moreover, lipid peroxidation and DNA damage [14] which are formed in response to Cis administration are also reported [15, 16]. Genotoxicity induced by some antitumor agents into normal tissues might also result in secondary malignancies [17]. Several antioxidant agents, in particular, have been used to inhibit the formation of free radicals, and they can also be used to prevent or reduce the adverse effects caused by Cis [18]. There are many studies which demonstrated the use of antioxidants in mitigating the adverse effects related to Cis [1, 17, 19–21].

Naringenin-oxime (NOX) is a new derivative compound of naringenin (4,5,7-trihydroxyflavanone) which is a predominant flavanone in citrus fruits including oranges and grapefruit [22]. Several studies have reported that naringenin (NG) has so many protective effects such as anti-inflammatory, antioxidant, anticarcinogenic effects, a promoter to carbohydrate metabolism, immune system modulator, and neuroprotective effects [23–25]. It was also suggested that NG possesses antiproliferative, antiatherogenic, apoptotic, and neuroprotective impacts [26–28]. NG has lately been used as an inducing agent for the composition of alternative new derivatives. Özyürek et al. [29] and Türkkan et al. [30] composed, defined, and analyzed some antioxidant aspects of a novel NG derivative compound called naringenin-oxime (NOX) and reported that antioxidant features of NG dramatically enhance in its Ox form. NOX has more antigenotoxic and antioxidative potential than the main compound NG [31].

The aim of this study is to demonstrate the beneficial effect of NOX on cisplatin-induced liver and kidney injury. To date, no study has yet evaluated* in vivo* protective effect of NOX on Cis toxicity. We aimed to assess the possible preservative effects of NOX on cisplatin-mediated injury in a small animal study.

## 2. Materials and Methods

### 2.1. Materials

**Scheme 1:**
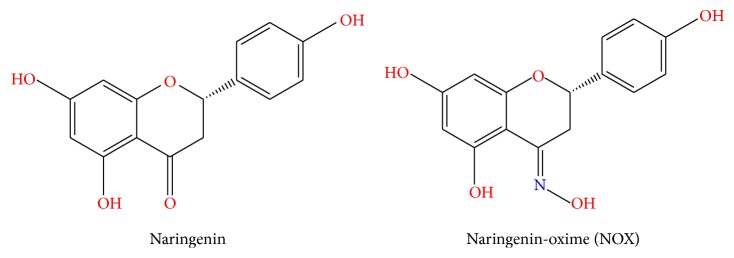


Cis (cis-diammineplatinum(II) dichloride) and chemicals were purchased from commercial suppliers (Sigma-Aldrich, Germany) and used as received. All reagents and solvents were of analytical reagent grade. NOX ((±)-2,3-dihydro-5,7-dihydroxy-2-(4-hydroxyphenyl)-4H-1-benzopyran-4-one oxime) was resynthesized as described in the literature (Scheme 1) [30].

### 2.2. Experimental Methods

Female and male Wistar albino rats (*n* = 35, 8 week-old), weighing 200–220 g, were taken from Dollvet Research Center (Şanlıurfa, Turkey). The rats were kept in a place which was environmentally controlled at constant temperature (21 ± 1°C) and humidity (70 ± 5%) with a 12 h light/dark periods until experiments. The rats were accustomed to the climate conditions for a week prior to study procedure and accessed freely to standard laboratory feed and water ad libitum. This research has the approval of Ethics Review Committee for Ethics in Animal Experiments of the Dollvet Research Center and strictly followed standards for the Guide for the Care and Use of Laboratory Animals (Ethics Committee Decision Number: 2014/66).

Cis toxicity was induced by a single dose intraperitoneal injection (i.p.) of Cis (i.p., 7 mg/kg b.w.). All rats were randomly split up into five groups (each of which includes 7 rats) as follows: control group (0.1 ml of normal saline i.p.), Cis group (i.p.,7 mg/kg), NOX (i.p., 20 mg/kg b.w., for 10 days), NOX (i.p., 20 mg/kg b.w., for 10 days) + Cis (i.p., 7 mg/kg b.w.) cotreated, and NOX (i.p., 40 mg/kg b.w., for 10 days) + Cis (i.p., 7 mg/kg b.w.) cotreated. NOX administration was initiated two days prior to the Cis injection. NOX was solved in DMSO and diluted in saline. Final DMSO concentration was less than 1%.

### 2.3. Blood Collection and Isolation of Rat PBMC

After experimental process (10 days), blood samples were taken from each group while the rats were under anesthesia. After decapitation of the animals, blood samples were taken according to cardiopuncture method, and they were put into blood collection tubes with and without heparin. By the difference of density gradient, the peripheral blood mononuclear cells (PBMC) were isolated by a gradient medium (Hystopaque 1077). Cell viability was assessed by using trypan blue dye exclusion technique. The ratio of vital cells was at least 90% before comet assay.

### 2.4. Preparation of Kidney and Liver Tissue Homogenates

Kidney and liver tissues were rinsed with ice-cold PBS (phosphate buffered saline) and kept at −80°C until analysis. To produce 10% (w/v) homogenate, tissues were homogenized with ice-cold Tris-HCl buffer (0.15 M, pH 7.4) for 5 minutes by a manual homogenizer. Then the homogenate was centrifuged at 7.000 ×g for 15 min. The pellet was separated and the clear supernatant was used for the analysis. All procedures were performed at 4°C.

### 2.5. Measurement of Biochemical Parameters

Blood urea nitrogen (BUN) and creatine (CRE) levels were evaluated by relying on an enzymatic technique carried out by an autoanalyzer (Cobas Integra 800, Roche). Myeloperoxidase (MPO) activity of serum was tested by the method defined by Bradley et al. Catalase (CAT) activities in tissue homogenates and serum were analyzed by the assay of Aebi. The basis of the assay is associated with the rate constant,* k* (dimension: s^−1^), of H_2_O_2_ decomposition. Ceruloplasmin (CERP) activity was evaluated in accordance with the assay of Erel et al. In this assay, ferrous ion is oxidized to ferric ion by ceruloplasmin ferrum oxidase activity. The findings are communicated as U/L (unite/liter). Paraoxonase (PON) and arylesterase (ARY) activities were assessed in serum and liver homogenates with commercially available kits (Rel assay, Turkey) by using an autoanalyzer (Cobas Integra 800, Roche).

Glutathione (GSH) level was assessed through reaction with OPA (1 mg/ml o-phthaldialdehyde in methanol) following to the modified technique of Kanďár and Hájková [32]. GSH was used as a standard. GSH samples were assessed via microplate reader (Spectra max M5, USA), with excitation at 345 nm and emission at 425 nm. Results were expressed as nmol/ml and nmol/g in serum and in wet tissue, respectively.

Malondialdehyde (MDA) levels in the liver and kidney tissues and in serum were assessed following the technique defined by Ohkawa et al. [33]. ELISA plates were read by a microplate reader (Spectra max M5), at 532 nm. The results were obtained as nmol/ml in serum and nmol/g in wet tissue, respectively.

Total oxidant status (TOS) and total antioxidant status (TAS) were detected in serum and tissue homogenates by using commercially available kits (Rel Assay, Turkey) with an autoanalyzer ((Cobas Integra 800, Roche)). TOS and TAS results were presented in mmol H_2_O_2_ equivalent/L [34] and mmol Trolox equivalent/L, respectively [35]. The ratio of the TOS to the TAS revealed the oxidative stress index (OSI), which is used as an indicator for total oxidative stress [36].

### 2.6. Comet Assay

The alkaline single cell gel electrophorese analysis (comet assay) was used to study the potential preventive effects of the NOX on Cis-induced DNA damage in PBMC genotoxicity in rats. Comet assay was performed according to Singh et al. (1988) with slight modifications [31] as follows: approximately 2 × 10^4^ cells were suspended in low melting point agarose (LMA) (75 *μ*l of 1.0%) and stratified onto semifrozen slides previously covered with a slim stratum of normal melting point agarose (1.0%). Another stratum of 0.5% LMA was put over the second layer. The cells were dissolved for 2 h at 4°C in a solution (100 mM EDTA, 2.5 M NaCl, 10% DMSO, 1% Triton X-100, 10 mMTris, pH 10.0). Following dissolution, the slides were exposed to electrophoresis in buffers (0.3 M NaOH, 1 mM EDTA, pH 13.1) for 30 min. Then, the slides were neutralized within a Tris buffer (0.4 M Tris-HCl, pH 7.5). The slides were carefully dried at 25°C in an incubator and marked with ethidium bromide (10 *μ*g/ml in distilled water, 70 *μ*l/slide). The slides were screened by using fluorescence microscope (Leica DM 1000, Solms, Germany) imaging system. A hundred cells were randomly scored in each sample on a scale of 0–4 based on fluorescence beyond the nucleus. The used scale scores were as follows: 0, no tail; 1, comet tail, half the width of the nucleus; 2, comet tail equal to the width of the nucleus; 3, comet tail longer than the nucleus; and 4, comet twice the width of the nucleus. Scoring cells in this way have been shown to be as accurate and precise as using computerized image analysis [31].

### 2.7. Measurement of 8-OHdG in Serum

8-Hydroxydeoxyguanosine (8-OHdG) is one of the very significant signs of oxidant-induced DNA damage. Quantification of 8-hydroxy-2′-deoxyguanosine (8-OHdG) was done by using OxiSelect™ Oxidative DNA Damage ELISA Kit (Cell Biolabs, San Diego, CA). Protocol was followed as described in the manufacturer instructions.

### 2.8. Statistics

Data obtained from study samples were presented as means and standard deviation of means (SD) and analyzed by one-way analysis of variance (ANOVA). TUKEY post hoc test on the SPSS (11.5) program was performed. Differences among the average values according to *p* < 0.01 were evaluated to be statistically significant.

## 3. Results

### 3.1. MDA, GSH, and CAT Activities Were Significantly Altered by NOX

Malondialdehyde (MDA) level is commonly used as an indicator for measuring free radical induced by LPO. A statistically significant (*p* < 0.01) increase was detected in MDA levels of kidney, serum, and liver of rats administered with Cis when compared to the control group. On the contrary, administration of NOX in a dose-dependent way demonstrated a significant (*p* < 0.01) decrease in kidney, liver, and serum when compared to the Cis group (Table 1).

Glutathione (GSH) is a tripeptide antioxidant which decreases the toxic metabolite of xenobiotics with a detoxification mechanism. GSH levels of serum, kidney, and liver (*p* < 0.01) of Cis groups were observed lower than the control group. However, applying NOX in a dose-dependent way indicated higher GSH levels of serum, liver, and kidney when compared to the Cis groups (Table 1).

The effect of NOX on the CAT activities in serum, kidney, and liver was measured in terms of cisplatin-induced toxicity in rats and the results are presented in Table 1. Treatment with only NOX is not affected by the CAT levels of serum, kidney, and liver tissues compared with the control group. The NOX_20_- and NOX_40_-treated rats significantly (*p* < 0.01) increased CAT level in the kidney and liver tissue compared to the Cis group.

### 3.2. Effects of NOX on Total Oxidant Status (TOS), Antioxidant Status (TAS), and Oxidative Stress Index (OSI) Levels

TOS and TAS were analyzed in kidney, liver tissues, and serum of rats and the results are shown in Table 2. In the Cis group, TAS was observed lower (*p* < 0.01) in serum, liver and kidney tissues compared with the control group. Nevertheless, TOS and OSI levels were increased (*p* < 0.01) in serum, liver, and kidney according to the control group. On the contrary, applying NOX in a dose-dependent way caused an inversion in Cis-mediated changes of TAS and TOS activities.

### 3.3. Effects of NOX on Serum and Liver PON and ARYL Activities

PON and ARYL operations were evaluated in serum and liver homogenates. Cis elicited a statistically significant decrease in PON and ARYL activities in serum and liver (*p* < 0.01) compared with the control group. However, combination of Cis with NOX_40_ was able to create a significant increase of PON levels in serum and liver compared with the Cis group (*p* < 0.01) (Table 3). Combination of Cis with NOX_20_ and NOX_40_ increased Cis-induced ARYL of serum, too (*p* < 0.01) (Table 3).

### 3.4. Effects on Biochemical Parameters of Serum

Compared with the control group, Cis treatment drastically enhanced ALT, AST, BUN, and creatinine levels (*p* < 0.01). NOX treatments caused an important decrease on those variables in a dose-dependent way (Table 4). Moreover, MPO and CERP levels were observed to be high in cisplatin group. The findings showed that NOX treatments decreased MPO and CERP levels previously increased caused by the use of Cis.

### 3.5. Defense against Cisplatin-Mediated DNA Damage

**Figure 1:**
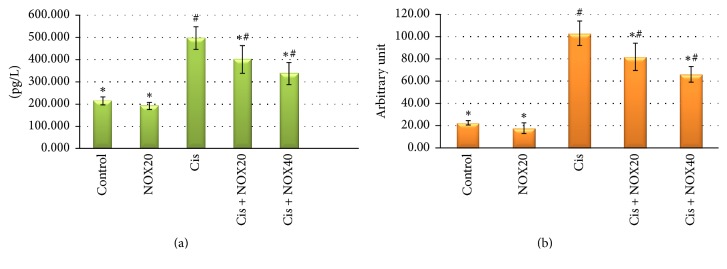
The effect of NOX on 8-OHdG (a) and DNA damage (comet assay) (b) levels in serum and PBMC of rats treated with Cis. Values are given as the means ± SD. There were no significant differences between the Cis and control groups in terms of any parameter (*p* > 0.01). ^#^*p* < 0.01, compared with the control group. ^*∗*^*p* < 0.01, groups in which Cis and NOX are treated together compared with the Cis group.

The comet assay and 8-OHdG results which indicate protective effect of NOX on cisplatin-mediated DNA damage are presented in Figure 1. Comet assay was conducted to analyze DNA damage in rat PBMCs. In the Cis group, 8-OHdG levels were observed to be high (497.1 ± 50.8 pg/L) compared with the control group (214.9 ± 17.8 pg/L) (*p* < 0.01). Also, 8-OHdG levels were 401.1 ± 62.2 and 337.9 ± 49, 7 pg/L (*p* < 0.01) in NOX_20_ and NOX_40_ treatments, respectively. These results suggest that treatment of NOX can significantly reduce 8-OHdG levels compared with the control group. Moreover, NOX prevented Cis-mediated change of 8-OHdG in a dose-depending way compared to the Cis group.

The protective effect of the NOX on rat PBMCs was also investigated using the comet assay. The comet assay was applied to determine the ratio of DNA in the tail. The results are showed in Figure 1. Only NOX treatment showed no genotoxic influence, and Cis treatment with NOX_20_ and NOX_40_ drastically decreased DNA migration compared to the Cis group (*p* < 0.01). Taking the genotoxicity into consideration, NOX treatment itself did not trigger DNA damage compared to the control group. There was an important increase in DNA fragmentation in Cis group when compared to the control group (Figure 1). Treatment of the animals with a compound of NOX along with Cis led to a statistically significant decline (*p* < 0.01) in DNA migration to the tail compared to the sole Cis treatment.

## 4. Discussion

Cis is one of the most widespread anticancer drugs used for the treatment of various cancer types. Cis administration might cause many unwelcome adverse effects, such as genotoxicity, nephrotoxicity, and hepatotoxicity that any of them may limit its clinical use. Efforts have been welcome to discover substances to trigger influential defense mechanisms against Cis toxicity. A number of experimental studies have analyzed several antioxidants such as naringenin, vitamin-E, bixin, vitamin-C, flavonoids, and carotenoid which were reported to demonstrate some protective effects in cisplatin-induced toxicity [17, 37, 38].

The objective of this study was to examine whether NOX can hamper some organ and tissue damage resulting from Cis. We have investigated whether it could increase the levels of MPO, TOS, DNA damage, markers of renal functions, serum liver enzymes, and MDA or enhance antioxidant defense mechanisms such as TAS, GSH, CAT, PON, and ARYL of serum, kidney, and liver tissues against Cis toxicity in rats.

Oxidative stress disrupts the equilibrium among the production and elimination of oxidants. This can be owing to an extreme level of oxidants produced in the body or weak antioxidant defense mechanisms that is mostly seen at chemotherapy regimens, especially with Cis treatments. In this respect, the present study assessed the overall effects of the use of Cis on oxidative and antioxidative status via different perspectives. OSI reveals the redox equilibrium among oxidation and antioxidation, and it has been used as the major indicator for body redox balance [39]. Separate evaluation of various oxidant molecules like hydroxyl radical and hydrogen peroxide (H_2_O_2_) is not practical; however their oxidant impacts are incremental. So we measured TOS in liver, kidney tissues, and serum in accordance with the previous descriptions by Erel [34]. Likewise, rather than individually determining antioxidant molecules, we measured TAS, according to the techniques of Erel, as well [35]. In previous studies, it was suggested that OSI could show the oxidative state more precisely than the sole levels of TOS or TAS [40, 41]. Hence, OSI could be regarded as an approximate indicator for identifying Cis-mediated genotoxicity, hepatotoxicity, nephrotoxicity, and preservation provided by NOX in subjects under Cis treatment. In the Cis-administrated group, the levels of TOS and OSI were dramatically enhanced compared with control and sole-NOX administrated groups, whereas TAS levels were clearly reduced when compared to the control group. However, applying NOX with Cis decreased the levels of TOS and OSI, while it enhanced TAS levels when compared with the Cis group. These results demonstrate that exposure to Cis caused an increase in oxidative stress in the tissues, and elevated oxidative stress was hampered by the NOX treatment.

The pharmacological mechanism of Cis in cancer treatment is related to its ability to coordinate with genomic DNA, in particular, the guanine residues. In spite of its success, cisplatin shows several drawbacks including increasing resistance and severe side effects, which are often associated with the variable repair mechanisms of nuclear DNA and off-target influences on certain cytoplasmic components [42–44].

Aforementioned free oxygen radicals may result in severe damage at nuclear DNA level. In this context, the extent of genotoxicity is assessed by 8-OHdG and comet assay in order to determine DNA damage. The biomarker, 8-OHdG, released secondary to DNA damage, is regarded as the most significant indicator of DNA damage [45, 46]. Hydroxyl radicals eliminate hydrogen from nucleic acids or react with double bonds, leading to 8-OHdG [47]. Altuner et al. [48] suggested that DNA damage is increased by following single dose Cis (5 mg/kg/d) in rat ovaries. Our results showed that 8-OHdG score is also a good indicator of tissue damage in Cis-induced genotoxicity in experimental rats.

Previous studies have suggested that DNA damage was decreased by antioxidant supplementation [17, 49], and the results of the present study supported these data. NOX exerted its antioxidant effects by reducing ROS-mediated 8-OHdG levels which indicates the prevention of oxidative DNA damage. This finding was the most important restorative effect of NOX treatment.

The protective effect of NOX on PBMCs of rats of which DNA damage was overt due to Cis induction was studied using comet assay. Findings of the comet assay demonstrated enhancements in the amount of DNA damage in Cis-administrated PBMCs. Treatment with NOX substantially diminished Cis-induced DNA damage in the comet assay. These results strongly demonstrate that NOX possess genoprotective efficiency.

In previous studies, it was suggested that AST, ALT, creatine, and BUN had been dramatically enhanced in Cis treated animals [49–51]. Similarly, the biochemical evaluation revealed increased levels of BUN AST, CRE, and ALT in the Cis group compared with the control group. We observed that their levels significantly decreased in administration of NOX at both concentrations (NOX_20_ and NOX_40_). The increased level of serum ALT and AST could be associated with the changed pattern of the liver cells. These are intracellular ALT and AST and are present in solely a small quantity in the blood serum. Harm to liver cells can cause escape into the serum owing to autolytic breakdown or cellular necrosis [52]. There are also some earlier studies suggesting escalated serum ALT and AST levels related to increased ROS [53, 54]. Enhanced levels of CRE and BUN can be attributed to retention of Cis in terminal collecting duct, the distal nephron of kidneys. This accumulation can cause tissue damage depending on exposure, time, and concentration [55]. In this study, it appears that treatment of rat with 20 and 40 mg/kg doses of NOX influentially protects the tissues against Cis-mediated hepatotoxicity and nephrotoxicity.

GSH is an important molecule and an omnipresent intracellular peptide. It has diverse functions such as maintaining cell antioxidant and oxidant balance, detoxification, modulation of cell proliferation, and maintenance of thiol status [38, 49, 51]. An important decrease in GSH levels generated by Cis shows an alteration in cellular redox state, suggesting that the cells can be more susceptible to ROS. This causes a decrease in efficacy of the antioxidant enzyme defense mechanism [50]. In the present study, GSH levels in the serum, liver, and kidney tissues of rats administered with Cis were lower than the control group. However, an increase in GSH levels in the serum, liver, and kidney tissues along with the supplementation of NOX suggests an enhancement in cellular response to oxidative stress. The impact of NOX on cellular GSH can be directly owing to antioxidant impacts or to the increased biosynthesis of GSH.

There are many studies indicating the relation between oxidative stress, MDA, and Cis-induced liver and kidney toxicity [49, 50, 53]. MDA is one of the basic indicators of oxidative harm and has been considered to take a significant part in the toxicity of several xenobiotics. Many pathophysiological systems have been put forward to clarify the enhanced MDA and reduced antioxidant levels in Cis toxicity [19, 40, 50, 53]. The data obtained from present study affirm that acute intoxication with Cis leads to an important elevation of MDA concentration in the liver. Prior treatment with NOX is quite influential in preventing the oxidative damage exerted by Cis. Alleviated oxidative stress has been defined by dramatically lower MDA concentration in serum, kidney, and liver. These results can also be associated with the significant role of NOX in hampering lipid peroxidation and in safeguarding the wholeness and functioning of tissues and cells.

PON is an enzyme which has an antioxidant feature. It was suggested that PON activity in tissue and/or serum significantly alters depending on oxidative stress [56]. In a study on rat liver tissues and human serum, Trudel et al. [57] reported that PON expression reduces the acute oxidative stress which is induced by the iron-ascorbate oxygen producing mechanism. This indicates that PON reacts to acute oxidative damage [58]. In the present study,* in vivo* preservative impacts of NOX against Cis toxicity were analyzed. PON and ARYL activities in both liver tissues and serum drastically reduced following the intraperitoneal Cis injection. On the other hand, applying NOX was found to dramatically hamper the reduction of PON enzyme activity of liver. This impact is possibly associated with the antioxidant feature of NOX.

In conclusion, this study shows that NOX compound has a preservative role in the mitigation of Cis-mediated hepatotoxicity, nephrotoxicity, and genotoxicity in rats. One possible reason of NOX-mediated preservation is that, before Cis administration, pretreatment with NOX could permit inception of free radicals produced by Cis prior to reaching DNA and causing damage. Moreover, a NOX-generated induction of DNA repair system might probably happen [31] Therefore, NOX could be an encouraging chemoprotective agent to tackle Cis-mediated toxicity and simultaneous treatment failure. It can also be helpful to prevent subsidiary damage in patients in a treatment regime involving Cis.

## Figures and Tables

**Table 1:** Effects of cisplatin (7 mg/kg), naringenin oxime (20 mg/kg), Cis + NOX_20_, and Cis + NOX_40_ on catalase activities, MDA, and GSH level in serum, kidney, and liver of rats.

	MDA			GSH			CATALASE		
	Serum (nmol/ml)	Kidney (nmol/g)	Liver (nmol/g)	Serum (nmol/ml)	Kidney (nmol/g)	Liver (nmol/g)	Serum (k/*µ*g protein)	Kidney (k/*µ*g protein)	Liver (k/*µ*g protein)
Control	10,7 ± 0,8^b^	10,9 ± 1,9^b^	8,03 ± 0,90^b^	7,3 ± 1,6^b^	7,7 ± 1,2^b^	13,13 ± 3,27^b^	9,18 ± 2,10^b^	2,8 ± 0,4^b^	5,76 ± 0,65^b^
Cis	19,3 ± 5,4^a^	19,6 ± 2,5^a^	15,56 ± 2,70^a^	3,4 ± 0,7^a^	4,8 ± 1,0^a^	8,23 ± 1,80^a^	5,85 ± 0,85^a^	1,2 ± 0,4^a^	3,03 ± 0,64^a^
Cis + NOX_20_	14,7 ± 2,9	16,8 ± 1,8^a^	11,22 ± 1,44^b^	5,1 ± 1,3	6,3 ± 1,4	9,99 ± 0,85	7,28 ± 1,83^a^	1,9 ± 0,4^a^	4,29 ±1,37
Cis + NOX_40_	12,5 ± 2,7^b^	12,1 ± 1,8^b^	9,85 ± 0,99^b^	6,3 ± 1,2^b^	7,1 ± 0,9^b^	12,11 ± 1,51^b^	8,02 ± 1,44	2,2 ± 0,3^b^	4,84 ± 1,22
NOX_20_	9,9 ± 1,6^b^	9,5 ± 2,3^b^	8,06 ± 1,78^b^	7,3 ± 0,7^b^	7,7 ± 0,9^b^	13,01 ± 1,43^b^	9,03 ± 2,19^b^	2,3 ± 0,4^b^	5,33 ± 0,68^b^
*p value*	0,000	0,000	*0,000*	0,000	0,000	*0,000*	0,000	0,000	*0,013*

Values are given as the means ± SD. There were no significant differences between the Cis and control groups in terms of any parameter (*p* > 0.01). ^a^*p* < 0.01, compared with the control group. ^b^*p* < 0.01, groups in which Cis and NOX are treated together compared with the Cis group.

**Table 2:** Effects of cisplatin (7 mg/kg), naringenin oxime (20 mg/kg), Cis + NOX_20_, and Cis + NOX_40_ on TOS, TAS, and OSI in serum, kidney, and liver of rats.

	TOS			TAS			OSI		
	Serum	Kidney	Liver	Serum	Kidney	Liver	Serum	Kidney	Liver
Control	10,5 ± 1,1^b^	8,3 ± 1,1^b^	7,38 ± 1,22^b^	1,28 ± 0,26^b^	0,87 ± 0,07^b^	0,72 ± 0,06^b^	0,88 ± 0,18^b^	0,96 ± 0,01^b^	1,03 ± 0,18^b^
Cis	18,1 ± 1,9^a^	13,4 ± 1,0^a^	11,72 ± 1,40^a^	0,68 ± 0,17^a^	0,61 ± 0,18^a^	0,43 ± 0,06^a^	2,71 ± 0,81^a^	2,33 ± 0,51^ab^	2,78 ± 0,52^a^
Cis + NOX_20_	15,3 ± 2,9^ab^	11,4 ± 2,1	9,51 ± 1,17	0,97 ± 0,16	0,76 ± 0,09	0,66 ± 0,16	1,64 ± 0,50^b^	1,51 ± 0,27^b^	1,53 ± 0,47^b^
Cis + NOX_40_	16,9 ± 3,9^b^	10,1 ± 1,7^b^	8,51 ± 1,10^b^	1,13 ± 0,32^b^	0,89 ± 0,05^b^	0,81 ± 0,15^b^	1,54 ± 0,28^b^	1,14 ± 0,20^b^	1,06 ± 0,10^b^
NOX_20_	9,8 ± 2,5^b^	8,3 ± 1,5^b^	7,42 ± 1,42^b^	1,20 ± 0,19^b^	0,88 ± 0,1^b^	0,79 ± 0,19^b^	0,82 ± 0,2^b^	0,26 ± 0,21^b^	0,98 ± 0,25^b^
*p value*	0,000	0,000	*0,000*	0,000	0,000	*0,000*	0,000	0,000	*0,000*

Values are given as the means ± SD. There were no significant differences between the cis and control groups in terms of any parameter (*p* > 0.01). ^a^*p* < 0.01, compared with the control group. ^b^*p* < 0.01, groups in which Cis and NOX are treated together compared with the Cis group.

**Table 3:** Effects of cisplatin (7 mg/kg), naringenin oxime (20 mg/kg) (NOX_20_), Cis + NOX_20_, and Cis + NOX_40_ on paraoxonase (PON) and arylesterase (ARYL) in serum and liver of rats.

	PON		ARYL	
	Serum	Liver	Serum	Liver
Control	138,3 ± 29,9^b^	239,9 ± 46,6^b^	68,5 ± 4,7^b^	111,4 ± 21,0^b^
Cis	114,0 ± 21,6^a^	126,9 ± 23,6^a^	59,9 ± 3,4^a^	72,3 ± 19,9^a^
Cis + NOX_20_	107,0 ± 51,5	157,1 ± 33,1	63,7 ± 12,5^b^	87,8 ± 21,9
Cis + NOX_40_	130,9 ± 16,1^b^	198,7 ± 25,8^b^	69,7 ± 10,8^b^	103,0 ± 13,9
NOX_20_	111,9 ± 45,6^b^	236,4 ± 34,8^b^	67,2 ± 5,9^b^	107,8 ± 6,2
*p value*	0,000	*0,000*	0,000	*0,001*

Values are given as the means ± SD. There were no significant differences between the cis and control groups in terms of any parameter (*p* > 0.01). ^a^*p* < 0.01, compared with the control group. ^b^*p* < 0.01, groups in which Cis and NOX are treated together compared with the Cis group.

**Table 4:** Effect of naringenin oxime (NOX) on serum liver enzymes, renal function markers, myeloperoxidase (MPO), and ceruloplasmin (CERP) of cisplatin induced rat.

	BUN (mg/dl)	Cre (mg/dl)	ALT (U/L)	AST (U/L)	MPO (U/g)	CERP
Control	38,69 ± 6,7^b^	0,28 ± 0,05^b^	35,4 ± 6,6^b^	73,3 ± 4,15^b^	27,2 ± 1,6	599,9 ± 41,1
Cis	64,11 ± 5,2^a^	0,60 ± 0,08^a^	67,3 ± 8,3^a^	144,2 ± 9,10^a^	30 ± 2,8	666,9 ± 86,6
Cis + NOX_20_	55,67 ± 5,6^a^	0,46 ± 0,05^ab^	52,6 ± 9,3^a^	105,3 ± 18,4^ab^	28,8 ± 2,8	601,6 ± 95,4
Cis + NOX_40_	47,30 ± 4,5^b^	0,38 ± 0,09^b^	42,9 ± 8,1^b^	86,7 ± 12,5^b^	28 ± 5,6	624,0 ± 30,7
NOX_20_	37,81 ± 6,3^b^	0,30 ± 0,08^b^	37,2 ± 6,5^b^	73,5 ± 10,2^b^	24,4 ± 4	597,4 ± 183,0
*p value*	0,000	0,000	0,000	0,000	0,600	0,685

Values are given as the means ± SD. There were no significant differences between the cis and control groups in terms of any parameter (*p* > 0.01). ^a^*p* < 0.01, compared with the control group. ^b^*p* < 0.01, groups in which Cis and NOX are treated together compared with the Cis group.
